# A Current Overview of Two Viroids That Infect Chrysanthemums: *Chrysanthemum stunt viroid* and *Chrysanthemum chlorotic mottle viroid*

**DOI:** 10.3390/v5041099

**Published:** 2013-04-17

**Authors:** Won Kyong Cho, Yeonhwa Jo, Kyoung-Min Jo, Kook-Hyung Kim

**Affiliations:** Department of Agricultural Biotechnology, Plant Genomics and Breeding Institute, Institute for Agriculture and Life Sciences, College of Agriculture and Life Sciences, Seoul National University, Seoul 151-921, Korea; E-Mails: wonkyong@gmail.com (W.K.C.); yeonhwajo@gmail.com (Y.J.); jokyoungmin@gmail.com (K.-M.J.)

**Keywords:** chrysanthemum, *Chrysanthemum stunt viroid* (CSVd), *Chrysanthemum chlorotic mottle viroid* (CChMVd), host-viroid interaction, viroid

## Abstract

The chrysanthemum (*Dendranthema* X *grandiflorum*) belongs to the family *Asteraceae* and it is one of the most popular flowers in the world. Viroids are the smallest known plant pathogens. They consist of a circular, single-stranded RNA, which does not encode a protein. Chrysanthemums are a common host for two different viroids, the *Chrysanthemum stunt viroid* (CSVd) and the *Chrysanthemum chlorotic mottle viroid* (CChMVd). These viroids are quite different from each other in structure and function. Here, we reviewed research associated with CSVd and CChMVd that covered disease symptoms, identification, host range, nucleotide sequences, phylogenetic relationships, structures, replication mechanisms, symptom determinants, detection methods, viroid elimination, and development of viroid resistant chrysanthemums, among other studies. We propose that the chrysanthemum and these two viroids represent convenient genetic resources for host–viroid interaction studies.

## 1. Introduction

The chrysanthemum (*Dendranthema* X *grandiflorum*) is a member of the family *Asteraceae*, and it is one of the popular flowers in the world. The international market for cut and potted chrysanthemums is increasing, and chrysanthemums in many European and Asian countries are commercially very important for the floral industry [1]. Several pathogens, including viruses, viroids, and phytoplasma, cause serious diseases in chrysanthemums. To date, nine viruses and two viroids are known to infect chrysanthemums [2]. 

Viroids are the smallest known plant pathogens. They consist of a circular, single-stranded RNA, which does not encode a protein. Viroid RNAs range from 246 to 401 bases [3,4]. Viroids traffic from cell to cell via plasmodesmata [5]. To date, over 30 species of viroids have been reported; these can be divided into two families, the *Pospiviroidae* and the *Avsunviroidae* [6]. The family *Pospiviroidae* includes five genera, such as *Apscaviroid*, *Cocadviroid*, *Coleviroid*, *Hostuviroid*, and *Pospiviroid*. So far, ten species including *Chrysanthemum stunt viroid* (CSVd) and *Potato spindle tuber viroid* (PSTVd) are members of the genus *Pospiviroid* [7]. The genomic RNA of members of the family *Pospiviroidae*, which replicate in the nucleus, assumes rod-like or quasi-rod-like conformation in which, based on local sequence similarity, five domains have been proposed: the left terminal, pathogenicity, central, variable, and right-terminal domains [8]. Moreover, several conserved regions have been identified in the rod-like conformation, including the central conserved region (CCR), likely involved in replication [9,10,11,12], and the terminal conserved region (TCR) or the terminal conserved hairpin (TCH), which appear mutually exclusive [13,14,15]. The family *Avsunviroidae* is composed of three genera, such as *Avsunviroid*, *Elaviroid*, and *Pelamoviroid*. *Peach latent mosaic viroid* (PLMVd) and *Chrysanthemum chlorotic mottle viroid* (CChMVd) are members of the genus *Pelamoviroid*. They have highly branched structures with self-cleaving ribozymes, which are not present in the family *Pospiviroidae*. These viroids replicate in the host chloroplast [16]. 

Interestingly, chrysanthemums are a common host for two different viroids, the CSVd and the CChMVd, which are quite different from each other in structure and function (Table 1). Here, we summarize and discuss current research associated with CSVd and CChMVd, and we suggest that chrysanthemums and viroids would serve as convenient genetic resources for host-viroid interaction studies.

## 2. Symptoms, Isolation, Nucleotide Sequences, and Structures

Chrysanthemum stunt disease was reported in the early 1950s [17]. It causes light green young leaves, stunting, small leaves and flowers, and reduced rooting ability. Later, infectious material was first isolated from leaves of stunted chrysanthemum, after nucleic acid extraction, centrifugation in sucrose density gradient and electrophoresis in polyacrylamide gels demonstrated the presence of a low molecular weight RNA, which was different from the known PSTVd [17]. This novel viroid, associated with stunt disease in chrysanthemums, was distinct from other known viruses, and it was named CSVd [18]. The complete nucleotide sequence and secondary structure of CSVd was first determined in Australia in 1981 [19]. The identified CSVd comprised 356 bases and displayed about 69% sequence identity to PSTVd. Subsequently, another CSVd with 354 bases was sequenced [20]. 

**Table 1:** Characteristics of CSVd and CChMVd viroids.

Characteristics	CSVd	CChMVd
Disease	Chrysanthemum stunt	Chrysanthemum chlorotic mottle
Symptoms	Light green young leaves, chlorotic spots, stunting, small leaves and flowers, and decreased rooting ability	Yellow-green mottling, chlorosis, and dwarfed size
Family and genus	*Pospiviroidae*, *Pospiviroid*	*Avsunviroidae*, *Pelamoviroid*
Genome size	354–356 nt	398–401 nt
Replication method	Asymmetric rolling circle mechanism	Symmetric rolling circle mechanism with the hammerhead ribozymes
Replication localization	Nucleus	Chloroplast
Structure	Rod-like structure including central conserved region (CCR)	Branched conformation including hammerhead ribozymes
Transmission	Sap, grafting, and seed	Sap, grafting
Host	Chrysanthemums, *Petunia hybrida*, tomato, *Gynura aurantiaca*, *Ageratum*, dahlia, *Senecio*, *Vinca major*, *Argyranthemum frutescens* and many plants belonging to the families *Solanaceae* and *Asteraceae*	Restricted to chrysanthemums

**Figure 1:**
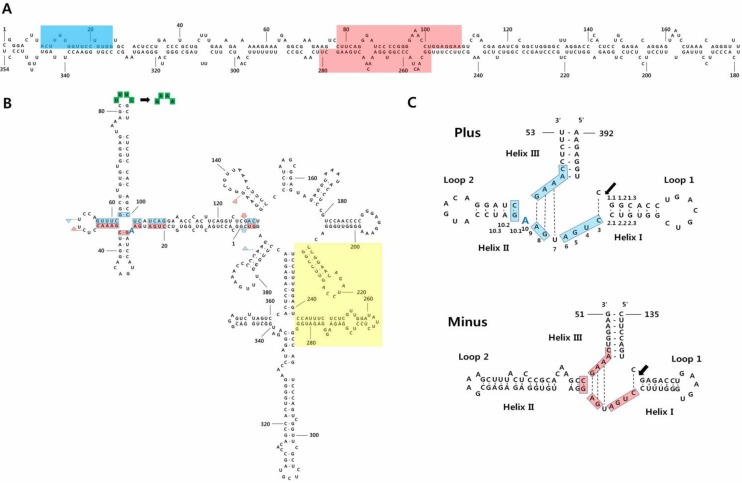
Predicted secondary structures of CSVd and CChMVd. (**A**) Predicted secondary structure of CSVd was adapted with permission from [22]. The central conserved region (CCR) is indicated by light-red shading and the terminal conserved region is indicated by light-blue shading; (**B**) Predicted secondary structure of CChMVd was adapted with permission from [28]. Plus and minus self-cleavage domains are delimited by flags, residues conserved in most natural hammerhead structures are boxed, and the self-cleavage sites are indicated by arrows. Light-blue shading and light-red shading in flags, boxes, and arrows refer to plus and minus polarities, respectively. The changes in the tetraloop delimited by positions 82–85 (UUUC to GAAA) that convert a symptomatic variant into non-symptomatic are shown with green-colored boxes [29]. The light-yellow square demarcates the domain that alternatively can form a kissing-loop interaction [30]; (**C**) Hammerhead structures of the plus and minus strands of CChMVd were adapted with permission from [28]. Residues conserved in most natural hammerhead structures are on light-blue shading and light-red shading in the plus and minus polarities, respectively, and the self-cleavage sites are indicated by arrows. Blue colored A indicates the position of the extra A in the CChMVd secondary structure and in its plus hammerhead structure. Numbering is done based on the previous study [28].

The structures and structural transitions of various viroids, including CSVd, have been determined with thermodynamic, kinetic, and hydrodynamic methods (Figure 1a) [21]. Recently, the sequences of three different CSVd isolates were determined from the US, China, and Australia. Both the US and Australian isolates were composed by several sequence variants, confirming the quasi-species nature of the viroid, but the Chinese isolate consisted of a single variant indicating a low molecular variability for CSVd [22]. Comparative analyses of the nucleotide sequences of 117 CSVd sequence variants revealed nucleotide variations at 103 sites scattered throughout the CSVd genome. However, it is not known whether these nucleotide changes are related to the species specificity of CSVd infections [23]; in addition, possible relationships between nucleotide changes and variant-specific pathogenicity have been recently questioned [23].

Chrysanthemum chlorotic mottle disease was first reported in 1967 in the cultivar “Yellow Delaware” in New York State [24]. This disease could be transmitted by grafting one chrysanthemum to another chrysanthemum cultivar. It causes yellow-green mottling, chlorosis, and dwarf symptoms, but some infected cultivars were asymptomatic [25]. Due to the low abundance of CChMVd in infected plants, compared to the abundance of CSVd, the complete, 398–401 nt sequence of CChMVd was only identified in 1997 (Figure 1b) [26]. Transcripts of CChMVd *in vitro* were also infectious and caused chlorotic mottle disease [26]. CChMVd can form hammerhead structures in both plus and minus strands, which self-cleave during *in vitro* and *in vivo* transcription (Figure 1c) [26]. The hammerhead structures of CChMVd display unique features, including an unpaired A residue after the conserved A9 residue in the plus self-cleaving domain, and an unusually long helix II in the minus one (Figure 1c). The predicted secondary structure of CChMVd is a very stable, branched conformation, which is similar to that of PLMVd. In contrast to viroids with a quasi-rod-like conformation, both CChMVd and PLMVd were insoluble in 2 M LiCl [26]. In one recent study, an *in vitro* transcript of CChMVd was inoculated to measure the mutation rate of CChMVd, which resulted in being the highest one ever reported in any biological entity [27].

## 3. Host Range

Due to the limited number of studies on viroids, it was thought that CSVd only infected chrysanthemums [31]. Initial reports showed that chrysanthemum plants could serve as host for both CSVd and CChMVd. Moreover, circular and linear forms of CSVd, purified from infected chrysanthemum plants, were infectious when inoculated into *Gynura aurantiaca* plants [32]. Also, a linear CSVd RNA, synthesized by *in vitro* transcription, could infect chrysanthemum plants and other plants that belong to the families *Solanaceae* and *Asteraceae*, without causing any disease symptoms [33]. Under natural conditions, infections of CSVd have been reported in *Petunia hybrida* [34], *Ageratum*, *Dahlia* [35], and *Senecio* (cineraria) [36]. CSVd was also identified in *Vinca major* with reverse transcriptase (RT)-PCR [37]. An infection of CSVd, identified with RT-PCR, was also reported in asymptomatic *Argyranthemum frutescens* (marguerite daisy) [38,39]. In contrast to the quite wide range identified for CSVd, the known host range of CChMVd is very restricted. For example, of 51 species and cultivars tested, CChMVd was infectious in only two chrysanthemum species [40].

In general, CSVd and CChMVd are mechanically transmitted through tools, like knives and scissors, used on infected chrysanthemums and then on healthy material for grafting and flower cutting. A previous study also demonstrated seed-borne transmission of CSVd in chrysanthemums; in that case, the ambient temperature during a cross could influence the rate of CSVd transmission to progeny [41]. However, CSVd could not be transmitted from infested soil [42], and no insect vector has been reported to transmit it.

## 4. Identification of the Two Viroids

Many large-scale screening studies have been performed to detect CSVd and CChMVd. For instance, a total of 2,480 chrysanthemum samples collected from six Australian states were tested for viroid and virus infections with enzyme-linked, immunosorbent assays (ELISAs) and cDNA probes [43]. As a result, CSVd has been found in mixed infection with other viruses [43]. 

CSVd is one of several quarantined pathogens in European countries, under order of the European Union’s Plant Health Directive (2000/29/EC) [44]. The European Food Safety Authority (EFSA) has released a Scientific Opinion on the assessment of the risk of solanaceous pospiviroids for the EU territory and the identification and evaluation of risk management options [45]. In Italy, to produce healthy chrysanthemum plants, phytosanitary measures, like RT-PCR and hybridization assays, were carried out to test for CSVd infections in 39 seasonal and 93 year-round chrysanthemums [1]. In Slovenia, 12 out of 200 chrysanthemum plants were found to be infected with CSVd [46]. Recently, a study reported the first identification of CSVd in Turkey; where two infected plants out of 154 chrysanthemums tested positive in an RT-PCR assay [47]. Moreover, CSVd was identified by RT-PCR in infected chrysanthemum plants in Egypt [48].

In Asian countries, CSVd and CChMVd have been reported in Japan, Korea, China, and India. Cultivated and wild chrysanthemums were tested for CSVd infections in Japan with RT-PCR [49]. Out of 89 samples, 80 samples were infected with CSVd. In addition, eight wild chrysanthemum species, which did not exhibit any CSVd symptoms, carried CSVd. Among 21 CSVd isolates, a total of five variants were revealed; these variants showed frequent mutations. 

In Akita Prefecture, Japan, CChMVd infections in chrysanthemums caused distinct yellow leaf mottling and necrosis. These symptoms were caused by CChMVd variants containing a UUUC tetraloop (Figure 1b) [50], a structural domain already identified as a specific CChMVd pathogenic determinant [26]. A previous study had detected CChMVd infections in various chrysanthemums grown for three years in Akita Prefecture, revealing that 20% of chrysanthemums were infected with CChMVd [51]. In natural conditions, chrysanthemums that were only infected with CChMVd variants with the UUUC tetraloop did not show any noticeable disease symptoms [51]. In this case, light and temperature may interfere with symptoms induced by variants containing the symptomatic domain. Furthermore, in Kyoto, CChMVd was also detected in cut chrysanthemums from Japan and the Netherlands; this indicated that CChMVd infections have spread over a wide range in Japan [52]. 

In Korea, CSVd was first identified in chrysanthemum cv. Chunkwang in 2001. The complete sequences for two isolates (K1 and K2) were determined [53]. Later, a total of 64 commercial chrysanthemum cultivars in Korea were tested for CSVd infections, and the infection rate of each cultivar ranged from 9.7% to 66.8% [54]. Furthermore, the complete sequence was reported for the CChMVd-SSHA6 viroid from Korea; this variant caused yellow spots and growth reduction in some infected cultivars [54].

In China, CChMVd was first reported in 2008. In 2010, 13 samples with mild chlorotic spots were collected, and all were infected with CChMVd [55]. In India, chrysanthemum cultivars were screened for CSVd infections with RT-PCR and DNA-RNA hybridization: 70% of cultivars were infected with CSVd [36]. 

## 5. Replication Mechanisms

Viroids replicate *in vivo* via a rolling circle mechanism, which requires a specific cleavage procedure that converts multimeric viroid copies into monomeric forms [56,57]. Viroids, including CSVd, in the family *Pospiviroidae* replicate in the nucleus via an asymmetric rolling circle mechanism [58]. In contrast, CChMVd and members of the family *Avsunviroidae* replicate via a symmetric rolling circle mechanism in the chloroplast, mediated by the hammerhead ribozyme. The hammerhead ribozyme in the family *Avsunviroidae* determines self-cleavage of oligomeric RNAs during viroid replication; replication is further catalyzed by RNA polymerase and RNA ligase [59]. 

The hammerhead ribozyme of CChMVd has been intensively studied. The plus hammerhead ribozyme of CChMVd contains an additional A (A10) residue between the conserved A9 and the quasi-conserved G10.1 residues (Figure 1c) [28]. The additional A10 residue causes a moderate decrease in the trans-cleaving rate, while substitution of A10 to C and A10 to G causes major detrimental effects (Figure 1c). In contrast, the A10 to U substitution increased the trans-cleaving rate by 3–4-fold. Some hammerheads display deviations from the consensus sequence because certain residues might be involved in unknown important functions, other than self-cleavage [28]. In addition, high-pressure experiments identified two different types of CChMVd hammerhead conformation that exhibit fast-cleaving and slow cleaving activities [60].

Interactions between loop 1 and loop 2 of CChMVd hammerheads in both polarity strands (and in most natural hammerheads) have been shown to play major roles in self-clearing activity of these ribozymes [30]. Moreover, site-directed mutagenesis, bioassays, and progeny analysis studies showed that a kissing-loop interaction is important for *in vitro* folding and viroid infectivity (Figure 1b) [61]. For instance, the introduction of a single mutation in the kissing loops led to low or no infectivity, but an introduction of second mutation restoring the kissing-loop interaction resulted in an infectious agent [61]. These results indicated that the loop-loop tertiary interactions are important for viroid infectivity and for the folding and catalytic activity of most natural hammerheads [30].

## 6. Symptom Determinants

Of the two viroids discussed here, CSVd is the more serious pathogen. It leads to reductions in the quality and quantity of chrysanthemum production. However, the sequence regions that confer the different symptoms are currently unknown. In contrast, the functional determinants for pathogenicity have been mapped in CChMVd. A previous study identified a nonpathogenic CChMVd strain that could protect against challenge inoculations with a pathogenic strain. Sequence analysis and site-directed mutagenesis have revealed that a substitution from UUUC to GAAA in the pathogenic CChMVd strain led to a nonpathogenic viroid with normal replication ability (Figure 1b). The identified pathogenicity determinant was located in a tetraloop of the predicted, branched conformation of CChMVd [62]. Later, the tetraloop of CChMVd, was further investigated with site-directed mutagenesis, bioassay, and analyses of the progenies [29]. The authors found that substitution of the tetraloop with a triloop or a pentaloop did not affect the infectivity of CChMVd. However, the thermodynamically stable GAAA tetraloop of the nonpathogenic CChMVd strain could not be replaced with other stable tetraloops of the UNCG family without causing functional changes. These data indicate that the sequence was the major factor that preserved the functional tetraloop motif, rather than the structure. After the introduction of a site-directed modified CChMVd variant into chrysanthemum, this nonpathogenic variant eventually evolved into a pathogenic CChMVd one, which acquired the UUUC tetraloop characteristics. This study provided evidence of the stronger biological fitness of the pathogenic CChMVd with the UUUC tetraloop, compared to that of the nonpathogenic CChMVd with the GAAA tetraloop. 

## 7. Detection Methods

Due to the fact that viroids have very low molecular weights, polyacrylamide gel electrophoresis (PAGE) was first used to detect viroid RNAs [63,64]. Molecular method to detect and quantify CSVd RNA in infected plants based on hybridization assays, where the viroid RNA hybridized to a ^32^P-labeled cDNA of CSVd, were also developed [65,66]. The ^32^P-labelled cDNA probe that targeted PSTVd was able to detect both PSTVd and CSVd [67]. In addition, several nonradioactive probes were developed, like photobiotin-labeled DNA probes, [68,69]. Both ^32^P-labeled and biotin-labeled cDNA probes were used and compared for the detection of CSVd and PSTVd [69]. The most sensitive method for detecting viroids is probably the RT-PCR-based approach. A previous study developed a simple and rapid method for nucleic acid extraction, without tissue homogenization, for detecting CSVd [70]. Typically, two different methods have been applied for detecting CSVd, either a combination of RT-PCR and hybridization, or a combination of RT-PCR and ELISA [39,70]. 

To identify several viroids or a novel viroid in a single host, several RT-PCR primer sets or digoxigenin-labeled polyprobes have been developed based on consensus sequences identified in all pospiviroids [71,72]. Also, a one-tube/one-step RT-PCR method was developed to detect seven viroids, including CSVd, belonging to four genera [73]. In addition, a multiplex, direct RT-PCR method was developed to detect CSVd and CChMVd in a small amount of plant tissues [74,75]. Furthermore, a previous study developed real-time PCR (TaqMan^®^) assays for detecting six viroids, including CSVd, infecting solanaceous hosts [44]. Recently, a multiplex RT-PCR primer set was developed to detect viruses and viroids, including *Chrysanthemum virus B*, *Tomato aspermy virus*, CSVd, and CChMVd in chrysanthemums [76]. These assays might be useful for detecting different viroids infecting the same host simultaneously. These methods could be applied to quarantine, certification, and screening purposes. 

## 8. Efforts to Eliminate Viroids from Infected Plants

Several attempts have been made to generate viroid-free plants. First, a long-term heat treatment at 35 °C for 14–37 weeks was proposed for treating CSVd-infected plants [77]. In contrast, a cold treatment was shown to be effective for eliminating CSVd in infected chrysanthemum plants [78]. Those authors grew CSVd-infected chrysanthemum plants at 5 °C for six months; subsequently, the meristem tips of cold-treated chrysanthemums were used to generate non-infected chrysanthemum plants [78]. Another study found that promoting vegetative propagation during a low-temperature period could eliminate CSVd from the infected chrysanthemums [42]. However, it was also shown that, after reducing the level of CSVd RNA in the plant during a low-temperature period, CSVd replication could again increase when the infected plants were grown at normal or high temperatures [79]. 

Recently, a Japanese research group established a new approach for obtaining CSVd-free chrysanthemums with leaf primordium-free, shoot apical meristems (LP-free SAMs). The attachment of LP-free SAMs to the root tips of CSVd-free chrysanthemums or cabbage resulted in the production of 14% and 3% CSVd-free plants, respectively [80]. A previous study generated CSVd-free and CSVd-infected plants by culturing different-sized SAMs dissected from the “Piato” plant; the authors reported that CSVd-infected plants flowered, even in the long-day condition [81]. These data suggested that CSVd might induce autonomous flowering in chrysanthemum, which is known to be a qualitative, short-day flowering plant [81]. In the same way, this research group has regenerated CChMVd-free chrysanthemums [52]. To date, the elimination of viroids from infected plants has remained a challenge. 

## 9. Identification and Development of Viroid-Resistant Chrysanthemum Cultivars

Most cultivated chrysanthemums are known to be infected with CSVd, which even if asymptomatic, could transfer CSVd to other susceptible cultivars in fields. Controlling CSVd with chemical treatments and culturing approaches is currently a challenging endeavor. Therefore, the ideal would be to find chrysanthemum cultivars resistant to CSVd, for developing new CSVd-resistant chrysanthemum lines. In a recent study, 35 chrysanthemum lines, including commercial cultivars, wild species, and interspecific hybrids, were screened to identify resistant plants by exposing uninfected plants to CSVd-infected plants with a grafting system [82]. RT-PCR was routinely performed to check for CSVd infections in the upper leaves of the scion every month after grafting. Of the 35 lines, the “Okayamaheiwa” cultivar showed strong resistance against CSVd. After crossing the “Okayamaheiwa” cultivar with two other susceptible cultivars, “Sei-elza” and “Anri,” 76 and 8 F1 progeny individuals were produced. Among the F1 progeny, 14 were not infected with CSVd, thus indicating that CSVd resistance was achieved in the first hybrid generation [82]. CSVd was not present in the SAMs or LPs of resistant chrysanthemum cultivars after grafting. Taking the absence of CSVd in the SAMs and LPs as a marker, 20 cultivars were identified as CSVd resistant out of 85 commercial cultivars tested; however, later grafting experiments showed that CSVd was detected in two of these candidate resistant cultivars [83]. 

Another previous study examined six cultivars, and identified a cultivar named “Utage,” which exhibited a reduced level of CSVd replication. A total of 67 cultivars, obtained from the self-pollination of “Utage,” were tested for CSVd resistance by RT-PCR. Of those, three plants (C7, A30, and A27) were found to have strong CSVd resistance [84]. 

Transgenic chrysanthemum lines were generated where a specific double-stranded RNA ribonuclease gene (*pac1*) of *Schizosaccharomyces pombe* was introduced with Agrobacterium-mediated transformation [85,86]. The generated transgenic plant, which expressed pac1, exhibited reduced levels of viroid infection and accumulation [85]. In addition, these plants were resistant to *Tomato spotted wilt virus* (TSWV). When hybrid lines were crossed with the transgenic line or with a wild chrysanthemum species, the progeny displayed either resistance or high susceptibility to TSWV infections. This study demonstrated the utility of *pac1* for achieving both viroid and virus resistance. 

## 10. Other Studies Associated with CSVd and CChMVd

Cross-protection can be defined as the interference in symptom expression generated by a previously inoculated viroid against subsequent infection by other strains of the same or a closely related viroid [87]. Five different viroids have been used for cross-protection studies in tomato and chrysanthemum plants [87]. Each viroid displayed different symptoms in chrysanthemum plants and, interestingly, three of them, CSVd, a mild PSTVd strain, and a severe PSTVd strain, could protect against *Citrus exocortis viroid* (CEVd); in contrast, CChMVd could not protect against the severe PSTV strain, CEVd, or CSVd, even though the challenge-inoculated viroid had replicated. Thus, viroid-infected plants showing attenuated symptoms might induce cross-protection against other viroids. Moreover it has been shown that non-symptomatic strain of CChMVd induces cross-protection against the symptomatic strain of the same viroid species [62].

Virus and viroid infections generate small interfering RNAs that interact with the RNA-induced silencing complex (RISC) of the host. A previous study demonstrated the presence of small RNAs (22 nt) derived from CSVd by northern blot analysis accumulating in similar amounts in different plants, regardless of differences in symptoms [88]. In addition, small interfering RNAs may be involved in the inhibition of enzyme activity; for example, the stunted phenotype of CSVd-infected plants might be associated with decreased levels of the plant growth hormone gibberelic acid (GA), due to disturbed GA 20-oxidase activity [89]. 

## 11. Future Directions in Host-Viroid Interaction Studies

Compared to studies on plant viruses, studies on viroids are very limited and mostly restricted to their detection and identification. In particular, host factors involved in viroid replication, pathogenicity, and movement have not been well characterized. For studying host–viroid interactions, chrysanthemum interactions with CSVd and CChMVd have several advantages. First, the two viroids are quite different from each other in replication mechanisms and structure. They belong to two different viroid families, but they do have a common host: chrysanthemums. Second, several protein-RNA interaction techniques will lead to the identification of chrysanthemum proteins that might function for both viroids, while others might be specific for either CSVd or CChMVd. In addition, next-generation sequencing-based approaches might provide information on the chrysanthemum transcriptome and how it is regulated by the two different viroids. Chrysanthemum plants are easily transformed, and creating transgenic plants is not difficult. However, there are several obstacles to using the chrysanthemum as a host, including the finding of a viroid-free, diploid chrysanthemum, because most chrysanthemums are infected with viroids and many are polyploid. 

Currently, our research group is working to establish a model system for host-viroid interactions with transcriptomic approaches. We anticipate that host transcript RNA profiling studies will reveal several host candidate genes involved in chrysanthemum-viroid interactions. Characterization of host gene functions will provide important information in understanding viroid disease processes, which may facilitate the development of new methods for their control. 
